# (*Z*)-Ethyl 3-(2,6-diisopropyl­anilino)but-2-enoate

**DOI:** 10.1107/S1600536810003260

**Published:** 2010-02-03

**Authors:** Manuel Amézquita-Valencia, Simón Hernández-Ortega, G. Alejandra Suárez-Ortiz, Armando Cabrera

**Affiliations:** a Instituto de Química, Universidad Nacional Autónoma de México, Circuito Exterior, Ciudad Universitaria, México 04510, Mexico

## Abstract

The title compound, C_18_H_27_NO_2_, crystallizes as the enamine form with *Z* geometry. The β-enamino­ester fragment forms a dihedral angle of 87.5 (1)° with the isopropyl­phenyl frame. The structure exhibits an intra­molecular N—H⋯O hydrogen bond. In addition, in the crystal, mol­ecules are linked by a centrosymmetric inter­molecular N—H⋯O hydrogen bond.

## Related literature

For methods used in the preparation of β-enamino­ketones and β-enamino­esters, see: Zhang & Yang (2009 ▶); Bartoli *et al.* (2004 ▶); Braibante *et al.* (2006 ▶). These compounds are used the preparation of key inter­mediates of pharmaceutical products (Michael *et al.*, 1999 ▶), amino­acids (Palmieri & Cimmerelli, 1996 ▶), peptides and alkaloids (David *et al.*, 1999 ▶). For our work on the synthesis of enamino­esters, see: Amézquita-Valencia *et al.* (2009 ▶).
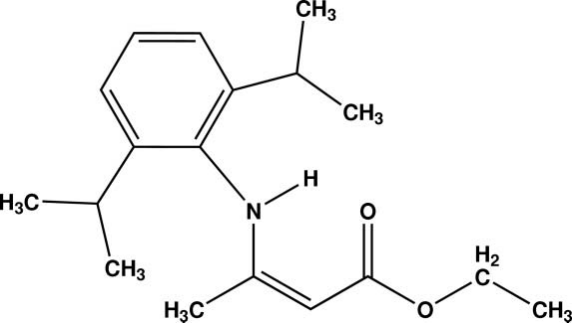


## Experimental

### 

#### Crystal data

C_18_H_27_NO_2_
*M* *_r_* = 289.41
Triclinic, 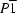

*a* = 8.4750 (17) Å
*b* = 8.8995 (18) Å
*c* = 11.956 (2) Å
α = 94.901 (3)°
β = 91.801 (3)°
γ = 101.255 (4)°
*V* = 880.1 (3) Å^3^
*Z* = 2
Mo *K*α radiation
μ = 0.07 mm^−1^
*T* = 298 K
0.29 × 0.21 × 0.05 mm

#### Data collection

Bruker SMART APEX CCD area-detector diffractometer
7319 measured reflections
3233 independent reflections
1574 reflections with *I* > 2σ(*I*)
*R*_int_ = 0.052

#### Refinement

*R*[*F* ^2^ > 2σ(*F* ^2^)] = 0.041
*wR*(*F* ^2^) = 0.085
*S* = 0.84
3233 reflections
199 parameters
1 restraint
H atoms treated by a mixture of independent and constrained refinement
Δρ_max_ = 0.11 e Å^−3^
Δρ_min_ = −0.10 e Å^−3^

### 

Data collection: *SMART* (Bruker, 1999 ▶); cell refinement: *SAINT* (Bruker, 1999 ▶); data reduction: *SAINT*; program(s) used to solve structure: *SHELXTL* (Sheldrick, 2008 ▶); program(s) used to refine structure: *SHELXTL*; molecular graphics: *SHELXTL*; software used to prepare material for publication: *SHELXTL*.

## Supplementary Material

Crystal structure: contains datablocks I, global. DOI: 10.1107/S1600536810003260/gw2075sup1.cif

Structure factors: contains datablocks I. DOI: 10.1107/S1600536810003260/gw2075Isup2.hkl

Additional supplementary materials:  crystallographic information; 3D view; checkCIF report

## Figures and Tables

**Table 1:** Selected Hydrogen-bond geometry (Å, °)

*D*—H⋯*A*	*D*—H	H⋯*A*	*D*⋯*A*	*D*—H⋯*A*
N1—H1⋯O1	0.89 (1)	2.02 (1)	2.7402 (18)	137 (1)
N1—H1⋯O1^i^	0.89 (1)	2.68 (1)	3.3371 (18)	132 (1)

Symmetry code: (i) 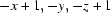.

**Fig. 1.**
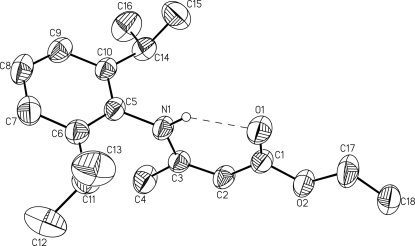
The Molecular structure of (I) with the atom numbering scheme. Displacement ellipsoids are drawn at the 40% probability level. H atoms bonded to C omitted. The intramolecular hydrogen bond is shown as a dashed line.

**Fig. 2.**
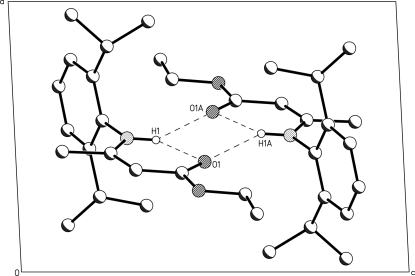
The centrosymmetric intermolecular N—H···O hydrogen bonding network in dashes lines. symmetry code 1-x, -y, 1-z.

## supplementary crystallographic information

### Comment

Several methods have been carried out in the β-enaminoketones and β-enaminoesters preparations, for instances; catalysis by silica-supported antimony (III) chloride (Zhang & Yang, 2009), the use of Lewis acids (Bartoli, *et al.* 2004) solid acids such as montmorillonite K10 (Braibante, *et al.* 2006). Is necessary to improve this reaction, due to the named compounds are important precursors in organic synthesis, these are particularly useful as they can be further transformed to key intermediates of several pharmaceutical products (Michael *et al.*, 1999). They have been utilized for the preparation of different important aminoacids (Palmieri *et al.*, 1996), peptides and alkaloids (David *et al.*, 1999). In continuation of our work in enaminoesters synthesis (Amézquita-Valencia, *et al.* 2009), we describe the structure of title compound (I) obtained using a Mexican bentonitic clay as catalist.

The structure with numbering scheme of the title compound is shown in fig. 1. The 2,6-diisopropylphenyl substituent is almost perpendicular to the β-enaminoester function (dihedral angle 87.5°), similar to described in ethyl 3-(2,6-dimethylphenylamino)but-2-enoate (Amézquita-Valencia, *et al.* 2009). A strong intramolecular N—H···O hydrogen bond is present in the structure, in addition, the molecules are linked by centrosymmetric intermolecular N—H···O hydrogen bond, the symmetry code is 1-x, -y 1-z, fig. 2.

### Experimental

Compound (I) was obtained following the procedure described in Amézquita-Valencia, *et al.* 2009. The product recrystallized from methanol. Yield: 92%, M.p. 326.3°

### Refinement

H atom on amine group was found in Fourier map and refined with *U*_iso_(H) = 1.2 UeqC(sp2). H on C atoms were placed in geometrically idealized positions [0.93 Å(CH) 0.96 Å(CH arom) and 0.97 Å (CH3)] tied to the parent atom with *U*_iso_(H) = 1.2 UeqC(sp2) and 1.5 UeqC(sp3) and refined using the riding model.

### Crystal data

**Table:**

C_18_H_27_NO_2_	*Z* = 2
*M**_r_* = 289.41	*F*(000) = 316
Triclinic, *P*1	*D*_x_ = 1.092 Mg m^−^^3^
Hall symbol: -P 1	Melting point: 326.3(2) K
*a* = 8.4750 (17) Å	Mo *K*α radiation, λ = 0.71073 Å
*b* = 8.8995 (18) Å	Cell parameters from 1942 reflections
*c* = 11.956 (2) Å	θ = 2.5–24.1°
α = 94.901 (3)°	µ = 0.07 mm^−^^1^
β = 91.801 (3)°	*T* = 298 K
γ = 101.255 (4)°	Prism, colorless
*V* = 880.1 (3) Å^3^	0.29 × 0.21 × 0.05 mm

### Data collection

**Table:**

Bruker SMART APEX CCD area-detector diffractometer	1574 reflections with *I* > 2σ(*I*)
Radiation source: fine-focus sealed tube	*R*_int_ = 0.052
graphite	θ_max_ = 25.4°, θ_min_ = 1.7°
Detector resolution: 0.83 pixels mm^-1^	*h* = −10→10
ω scans	*k* = −10→10
7319 measured reflections	*l* = −14→14
3233 independent reflections	

### Refinement

**Table:**

Refinement on *F*^2^	Primary atom site location: structure-invariant direct methods
Least-squares matrix: full	Secondary atom site location: difference Fourier map
*R*[*F*^2^ > 2σ(*F*^2^)] = 0.041	Hydrogen site location: inferred from neighbouring sites
*wR*(*F*^2^) = 0.085	H atoms treated by a mixture of independent and constrained refinement
*S* = 0.84	*w* = 1/[σ^2^(*F*_o_^2^) + (0.020*P*)^2^] where *P* = (*F*_o_^2^ + 2*F*_c_^2^)/3
3233 reflections	(Δ/σ)_max_ < 0.001
199 parameters	Δρ_max_ = 0.11 e Å^−^^3^
1 restraint	Δρ_min_ = −0.10 e Å^−^^3^

### Special details

**Table:**

Geometry. All esds (except the esd in the dihedral angle between two l.s. planes) are estimated using the full covariance matrix. The cell esds are taken into account individually in the estimation of esds in distances, angles and torsion angles; correlations between esds in cell parameters are only used when they are defined by crystal symmetry. An approximate (isotropic) treatment of cell esds is used for estimating esds involving l.s. planes.
Refinement. Refinement of *F*^2^ against ALL reflections. The weighted *R*-factor wR and goodness of fit *S* are based on *F*^2^, conventional *R*-factors *R* are based on *F*, with *F* set to zero for negative *F*^2^. The threshold expression of *F*^2^ > σ(*F*^2^) is used only for calculating *R*-factors(gt) etc. and is not relevant to the choice of reflections for refinement. *R*-factors based on *F*^2^ are statistically about twice as large as those based on *F*, and *R*- factors based on ALL data will be even larger.

### Fractional atomic coordinates and isotropic or equivalent isotropic displacement parameters (Å^2^)

**Table:**

	*x*	*y*	*z*	*U*_iso_*/*U*_eq_	
O1	0.40766 (16)	0.15127 (14)	0.48653 (10)	0.0730 (4)	
O2	0.30098 (15)	0.36079 (13)	0.46776 (9)	0.0683 (4)	
N1	0.49463 (18)	0.01679 (16)	0.28928 (11)	0.0554 (4)	
H1	0.4882 (18)	0.0192 (18)	0.3638 (8)	0.066*	
C1	0.3661 (2)	0.2415 (2)	0.42624 (15)	0.0556 (5)	
C2	0.3774 (2)	0.23743 (19)	0.30792 (14)	0.0574 (5)	
H2	0.3404	0.3129	0.2713	0.069*	
C3	0.4383 (2)	0.1311 (2)	0.24509 (14)	0.0521 (5)	
C4	0.4449 (2)	0.1348 (2)	0.12035 (14)	0.0821 (7)	
H4A	0.5552	0.1526	0.0999	0.123*	
H4B	0.3952	0.2161	0.0974	0.123*	
H4C	0.3885	0.0382	0.0838	0.123*	
C5	0.5595 (2)	−0.1002 (2)	0.22757 (13)	0.0508 (5)	
C6	0.7234 (2)	−0.0729 (2)	0.20876 (14)	0.0579 (5)	
C7	0.7848 (2)	−0.1914 (3)	0.15333 (15)	0.0697 (6)	
H7	0.8937	−0.1763	0.1392	0.084*	
C8	0.6859 (3)	−0.3301 (3)	0.11939 (15)	0.0710 (6)	
H8	0.7291	−0.4091	0.0842	0.085*	
C9	0.5242 (3)	−0.3537 (2)	0.13675 (14)	0.0638 (5)	
H9	0.4591	−0.4480	0.1118	0.077*	
C10	0.4557 (2)	−0.2397 (2)	0.19079 (13)	0.0529 (5)	
C11	0.8341 (2)	0.0779 (2)	0.24878 (18)	0.0793 (6)	
H11	0.7657	0.1534	0.2650	0.095*	
C12	0.9507 (3)	0.1411 (3)	0.1612 (2)	0.1274 (9)	
H12A	1.0269	0.0753	0.1486	0.191*	
H12B	1.0068	0.2428	0.1879	0.191*	
H12C	0.8915	0.1448	0.0920	0.191*	
C13	0.9264 (3)	0.0662 (3)	0.35821 (18)	0.1140 (8)	
H13A	0.8516	0.0412	0.4157	0.171*	
H13B	0.9972	0.1628	0.3807	0.171*	
H13C	0.9884	−0.0129	0.3471	0.171*	
C14	0.2780 (2)	−0.2639 (2)	0.21149 (15)	0.0665 (5)	
H14	0.2455	−0.1650	0.2053	0.080*	
C15	0.2454 (2)	−0.3071 (2)	0.33083 (17)	0.0971 (7)	
H15A	0.2766	−0.4035	0.3403	0.146*	
H15B	0.1326	−0.3164	0.3431	0.146*	
H15C	0.3064	−0.2286	0.3840	0.146*	
C16	0.1727 (2)	−0.3808 (2)	0.12593 (19)	0.1022 (8)	
H16A	0.1915	−0.3503	0.0516	0.153*	
H16B	0.0614	−0.3855	0.1412	0.153*	
H16C	0.1988	−0.4803	0.1311	0.153*	
C17	0.2874 (3)	0.3794 (2)	0.58714 (14)	0.0862 (7)	
H17A	0.3930	0.3940	0.6249	0.103*	
H17B	0.2202	0.2884	0.6120	0.103*	
C18	0.2146 (2)	0.5158 (2)	0.61480 (15)	0.0817 (6)	
H18A	0.2811	0.6051	0.5891	0.123*	
H18B	0.2064	0.5312	0.6947	0.123*	
H18C	0.1092	0.4994	0.5785	0.123*	

### Atomic displacement parameters (Å^2^)

**Table:**

	*U*^11^	*U*^22^	*U*^33^	*U*^12^	*U*^13^	*U*^23^
O1	0.1105 (11)	0.0678 (9)	0.0517 (8)	0.0418 (8)	0.0065 (7)	0.0118 (7)
O2	0.1025 (11)	0.0614 (8)	0.0497 (8)	0.0382 (8)	0.0095 (7)	0.0023 (6)
N1	0.0797 (11)	0.0503 (9)	0.0418 (8)	0.0262 (8)	0.0075 (8)	0.0038 (8)
C1	0.0659 (14)	0.0490 (12)	0.0539 (12)	0.0173 (11)	0.0035 (10)	0.0014 (10)
C2	0.0800 (14)	0.0495 (11)	0.0487 (11)	0.0265 (10)	0.0032 (10)	0.0070 (9)
C3	0.0637 (13)	0.0487 (11)	0.0461 (11)	0.0158 (10)	0.0014 (9)	0.0067 (9)
C4	0.1282 (19)	0.0817 (15)	0.0491 (11)	0.0482 (14)	0.0115 (11)	0.0131 (11)
C5	0.0670 (14)	0.0497 (12)	0.0405 (10)	0.0222 (11)	0.0045 (10)	0.0052 (9)
C6	0.0666 (15)	0.0558 (13)	0.0546 (11)	0.0179 (12)	0.0060 (10)	0.0089 (10)
C7	0.0696 (15)	0.0803 (15)	0.0657 (13)	0.0283 (14)	0.0127 (11)	0.0091 (12)
C8	0.0917 (18)	0.0764 (16)	0.0537 (12)	0.0419 (14)	0.0057 (12)	−0.0036 (11)
C9	0.0837 (16)	0.0548 (12)	0.0541 (12)	0.0210 (12)	−0.0033 (11)	−0.0027 (10)
C10	0.0703 (15)	0.0498 (12)	0.0429 (10)	0.0222 (11)	0.0012 (10)	0.0050 (9)
C11	0.0711 (15)	0.0695 (15)	0.0960 (17)	0.0116 (13)	0.0051 (13)	0.0055 (13)
C12	0.112 (2)	0.124 (2)	0.139 (2)	−0.0138 (17)	0.0189 (18)	0.0496 (18)
C13	0.117 (2)	0.1073 (19)	0.0989 (18)	−0.0166 (16)	−0.0228 (16)	0.0004 (15)
C14	0.0711 (15)	0.0559 (13)	0.0741 (14)	0.0173 (11)	0.0021 (11)	0.0060 (11)
C15	0.0893 (18)	0.1064 (18)	0.0986 (17)	0.0148 (14)	0.0304 (14)	0.0286 (15)
C16	0.0800 (17)	0.0877 (17)	0.132 (2)	0.0130 (14)	−0.0184 (15)	−0.0127 (15)
C17	0.137 (2)	0.0852 (15)	0.0495 (12)	0.0540 (15)	0.0167 (12)	0.0017 (11)
C18	0.1002 (17)	0.0771 (15)	0.0726 (14)	0.0309 (13)	0.0199 (12)	−0.0032 (11)

### Geometric parameters (Å, °)

**Table:**

O1—C1	1.2173 (18)	C11—C13	1.525 (3)
O2—C1	1.3543 (18)	C11—C12	1.529 (2)
O2—C17	1.4337 (18)	C11—H11	0.9800
N1—C3	1.3439 (19)	C12—H12A	0.9600
N1—C5	1.4310 (19)	C12—H12B	0.9600
N1—H1	0.894 (8)	C12—H12C	0.9600
C1—C2	1.419 (2)	C13—H13A	0.9600
C2—C3	1.350 (2)	C13—H13B	0.9600
C2—H2	0.9300	C13—H13C	0.9600
C3—C4	1.497 (2)	C14—C16	1.525 (2)
C4—H4A	0.9600	C14—C15	1.529 (2)
C4—H4B	0.9600	C14—H14	0.9800
C4—H4C	0.9600	C15—H15A	0.9600
C5—C6	1.392 (2)	C15—H15B	0.9600
C5—C10	1.402 (2)	C15—H15C	0.9600
C6—C7	1.392 (2)	C16—H16A	0.9600
C6—C11	1.510 (2)	C16—H16B	0.9600
C7—C8	1.371 (3)	C16—H16C	0.9600
C7—H7	0.9300	C17—C18	1.483 (2)
C8—C9	1.370 (2)	C17—H17A	0.9700
C8—H8	0.9300	C17—H17B	0.9700
C9—C10	1.388 (2)	C18—H18A	0.9600
C9—H9	0.9300	C18—H18B	0.9600
C10—C14	1.510 (2)	C18—H18C	0.9600
			
C1—O2—C17	116.95 (14)	C11—C12—H12A	109.5
C3—N1—C5	125.68 (14)	C11—C12—H12B	109.5
C3—N1—H1	113.3 (10)	H12A—C12—H12B	109.5
C5—N1—H1	121.1 (10)	C11—C12—H12C	109.5
O1—C1—O2	121.97 (16)	H12A—C12—H12C	109.5
O1—C1—C2	126.24 (17)	H12B—C12—H12C	109.5
O2—C1—C2	111.79 (16)	C11—C13—H13A	109.5
C3—C2—C1	124.19 (16)	C11—C13—H13B	109.5
C3—C2—H2	117.9	H13A—C13—H13B	109.5
C1—C2—H2	117.9	C11—C13—H13C	109.5
N1—C3—C2	122.83 (15)	H13A—C13—H13C	109.5
N1—C3—C4	116.49 (15)	H13B—C13—H13C	109.5
C2—C3—C4	120.68 (15)	C10—C14—C16	113.93 (16)
C3—C4—H4A	109.5	C10—C14—C15	111.14 (15)
C3—C4—H4B	109.5	C16—C14—C15	110.36 (17)
H4A—C4—H4B	109.5	C10—C14—H14	107.0
C3—C4—H4C	109.5	C16—C14—H14	107.0
H4A—C4—H4C	109.5	C15—C14—H14	107.0
H4B—C4—H4C	109.5	C14—C15—H15A	109.5
C6—C5—C10	122.50 (16)	C14—C15—H15B	109.5
C6—C5—N1	118.98 (17)	H15A—C15—H15B	109.5
C10—C5—N1	118.49 (17)	C14—C15—H15C	109.5
C7—C6—C5	117.78 (18)	H15A—C15—H15C	109.5
C7—C6—C11	120.22 (19)	H15B—C15—H15C	109.5
C5—C6—C11	121.99 (17)	C14—C16—H16A	109.5
C8—C7—C6	120.58 (19)	C14—C16—H16B	109.5
C8—C7—H7	119.7	H16A—C16—H16B	109.5
C6—C7—H7	119.7	C14—C16—H16C	109.5
C9—C8—C7	120.76 (18)	H16A—C16—H16C	109.5
C9—C8—H8	119.6	H16B—C16—H16C	109.5
C7—C8—H8	119.6	O2—C17—C18	108.30 (15)
C8—C9—C10	121.37 (19)	O2—C17—H17A	110.0
C8—C9—H9	119.3	C18—C17—H17A	110.0
C10—C9—H9	119.3	O2—C17—H17B	110.0
C9—C10—C5	116.97 (17)	C18—C17—H17B	110.0
C9—C10—C14	122.14 (18)	H17A—C17—H17B	108.4
C5—C10—C14	120.88 (16)	C17—C18—H18A	109.5
C6—C11—C13	111.31 (17)	C17—C18—H18B	109.5
C6—C11—C12	113.78 (18)	H18A—C18—H18B	109.5
C13—C11—C12	110.29 (19)	C17—C18—H18C	109.5
C6—C11—H11	107.0	H18A—C18—H18C	109.5
C13—C11—H11	107.0	H18B—C18—H18C	109.5
C12—C11—H11	107.0		
			
C17—O2—C1—O1	1.9 (2)	C7—C8—C9—C10	−1.2 (3)
C17—O2—C1—C2	−178.36 (16)	C8—C9—C10—C5	−0.6 (2)
O1—C1—C2—C3	−0.6 (3)	C8—C9—C10—C14	−179.51 (16)
O2—C1—C2—C3	179.68 (17)	C6—C5—C10—C9	1.9 (2)
C5—N1—C3—C2	179.47 (17)	N1—C5—C10—C9	−176.47 (13)
C5—N1—C3—C4	0.0 (3)	C6—C5—C10—C14	−179.16 (14)
C1—C2—C3—N1	0.2 (3)	N1—C5—C10—C14	2.5 (2)
C1—C2—C3—C4	179.67 (17)	C7—C6—C11—C13	−79.3 (2)
C3—N1—C5—C6	88.8 (2)	C5—C6—C11—C13	99.3 (2)
C3—N1—C5—C10	−92.8 (2)	C7—C6—C11—C12	46.0 (2)
C10—C5—C6—C7	−1.4 (2)	C5—C6—C11—C12	−135.40 (19)
N1—C5—C6—C7	176.92 (14)	C9—C10—C14—C16	−27.7 (2)
C10—C5—C6—C11	179.97 (15)	C5—C10—C14—C16	153.41 (16)
N1—C5—C6—C11	−1.7 (2)	C9—C10—C14—C15	97.75 (19)
C5—C6—C7—C8	−0.4 (2)	C5—C10—C14—C15	−81.16 (19)
C11—C6—C7—C8	178.25 (17)	C1—O2—C17—C18	179.70 (16)
C6—C7—C8—C9	1.7 (3)		

### Hydrogen-bond geometry (Å, °)

**Table:**

*D*—H···*A*	*D*—H	H···*A*	*D*···*A*	*D*—H···*A*
N1—H1···O1	0.89 (1)	2.02 (1)	2.7402 (18)	137 (1)
N1—H1···O1^i^	0.89 (1)	2.68 (1)	3.3371 (18)	132 (1)

Symmetry codes: (i) −*x*+1, −*y*, −*z*+1.
